# Severity assessment in mice subjected to carbon tetrachloride

**DOI:** 10.1038/s41598-020-72801-1

**Published:** 2020-09-25

**Authors:** Lisa Ernst, Leonie Zieglowski, Mareike Schulz, Michaela Moss, Marco Meyer, Ralf Weiskirchen, Rupert Palme, Melanie Hamann, Steven R. Talbot, René H. Tolba

**Affiliations:** 1 Institute for Laboratory Animal Science and Experimental Surgery, Faculty of Medicine, RWTH, Aachen International University, Aachen, Germany; 2 Institute of Molecular Pathobiochemistry, Experimental Gene Therapy and Clinical Chemistry (IFMPEGKC), RWTH University Hospital Aachen, Aachen, Germany; 3 Department of Biomedical Sciences, University of Veterinary Medicine, Vienna, Austria; 4 Institute of Pharmacology and Toxicology, Faculty of Veterinary Medicine, Justus Liebig University, Giessen, Germany; 5 Institute for Laboratory Animal Science, Hannover Medical School, Hanover, Germany

**Keywords:** Zoology, Gastroenterology, Medical research

## Abstract

The Directive 2010/63 EU requires classifying burden and severity in all procedures using laboratory animals. This study evaluated the severity of liver fibrosis induction by intraperitoneal carbon tetrachloride (CCl_4_) injections in mice. 29 male C57BL/6N mice were treated three times per week for 4 weeks with an intraperitoneal injection (50 µl) of either 0.6 ml/kg body weight CCl_4_-vehicle solution, germ oil (vehicle-control) or handling only. Severity assessment was performed using serum analysis, behavioral tests (open field test, rotarod, burrowing and nesting behavior), fecal corticosterone metabolite (FCM) measurement, and survival. The most significant group differences were noticed in the second week of treatment when the highest AST (1463 ± 1404 vs. 123.8 ± 93 U/L, p < 0.0001) and nesting values were measured. In addition, respective animals showed lower moving distances (4622 ± 1577 vs. 6157 ± 2060 cm, p < 0.01) and velocity in the Open field, identified as main factors in principal component analysis (PCA). Overall, a 50% survival rate was observed within the treatment group, in which the open field performance was a good tracer parameter for survival. In summary, this study demonstrates the feasibility of assessing severity in mice using behavioral tests and highlight the open field test as a possible threshold parameter for risk assessment of mortality.

## Introduction

With the implementation of the European Directive 2010/63/EU on the protection of animals used for scientific purposes, Article 15 requires a prospective assessment of the severity of each procedure in a scientific experiment and a severity classification, which may be either "non-recovery", "mild", "moderate" or "severe"^1^.

At the same time, to achieve the implementation of the EU Directive 2010/63 in their Member States, the aim of scientific experiments using experimental animals should not only characterize the various methods in terms of their severity but also ensure that the methods are standardized, regardless of application. To this end, it is necessary to provide detailed and standardized operating procedures, both for the experiments to be carried out and for the performance of various tests^2^. However, since most of the animal species used in biomedical research are flight animals, these will not express their harm by audible vocalization and will try to avoid any display of suffering^3^. As a consequence, researchers have to assign different degrees of severity to diverse procedures, always according to the behavioral characteristics of the respective species.

In the research of liver diseases, different techniques for the induction of liver fibrosis in mice are presently used. The application of CCl_4_ to induce liver damage, which has been introduced in the early 1950s^4^, is still one of the most commonly used approaches for chemical-mediated induction of liver fibrosis in mice or rats^5^. CCl_4_ administration, given via intraperitoneal (i.p.) injection two to three times a week over 4–6 weeks in most protocols, is a model that is leading to a representative and reproducible fibrosis^5^, which can also be reversible after discontinuation of the administration^6^. Thus, this model is often used in the research of fibrosis development as well as in the investigation of liver repair mechanisms, underlining the scientific importance of this model. The aim of this research project was to evaluate the severity of induction of liver fibrosis in mice within the periodic administration of CCl_4_ in a 4-week i.p. administration model.

## Results

**Figure 1:**
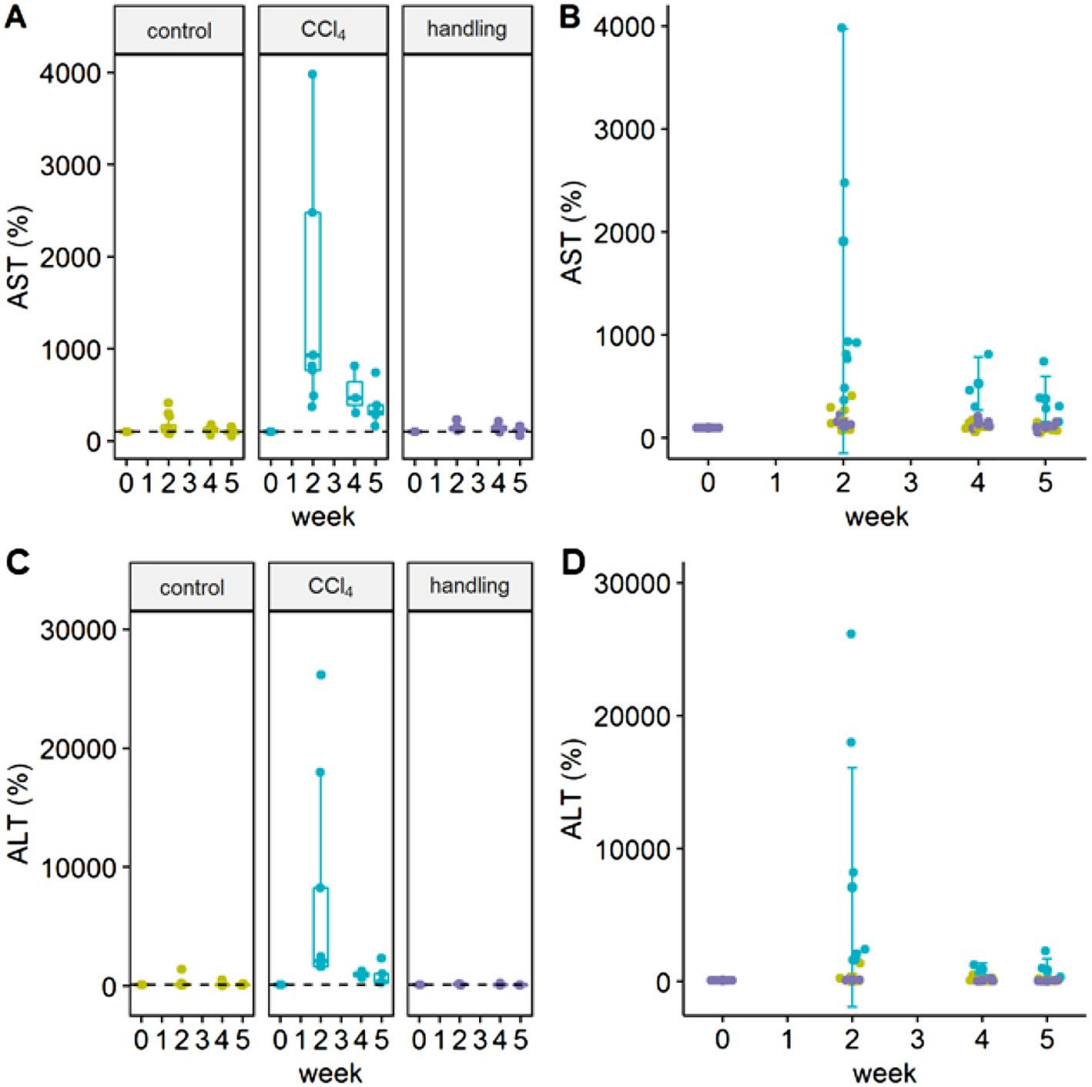
Serum levels of AST and ALT in %. (**A**) Serum values of AST are depicted as box plots with an indication of the median value. Whiskers are determined using the Tukey method (1.5 × IQR). (**B**) Individual AST values in the three experimental groups are given. Type III ANOVA F(2; 85) = 7.6130; week 2: CCl_4_ vs control; ***p < 0.001, p_adj_ < 0.0001; week 2: CCl_4_ vs handling; ***p < 0.001 p_adj_ < 0.0001. (**C**) Serum values of ALT with indication of the median value (**D**) Individual ALT values of all experimental groups. Type III ANOVA F(2;20.54) = 3.6966; week 2: CCl_4_ vs. Control; ***p < 0.001; p_adj_ < 0.0001; week 2: CCl_4_ vs handling; ***p < 0.001 p_adj_ < 0.0001. The graphs were generated using the R software version (3.6.2) (R Core Team (2017). R: A language and environment for statistical computing. R Foundation for Statistical Computing, Vienna, Austria. https://www.R-project.org/).

Serum levels of aspartate aminotransferase (AST; Fig. 1A,B) as well as alanine aminotransferase levels (ALT; Fig. 1C,D) are significantly increased in the treatment group at the time of the second week after starting CCl_4_ administration. Bilirubin levels were not elevated and showed equivalent serum levels in the treatment, control or handling groups (data not shown).

**Figure 2:**
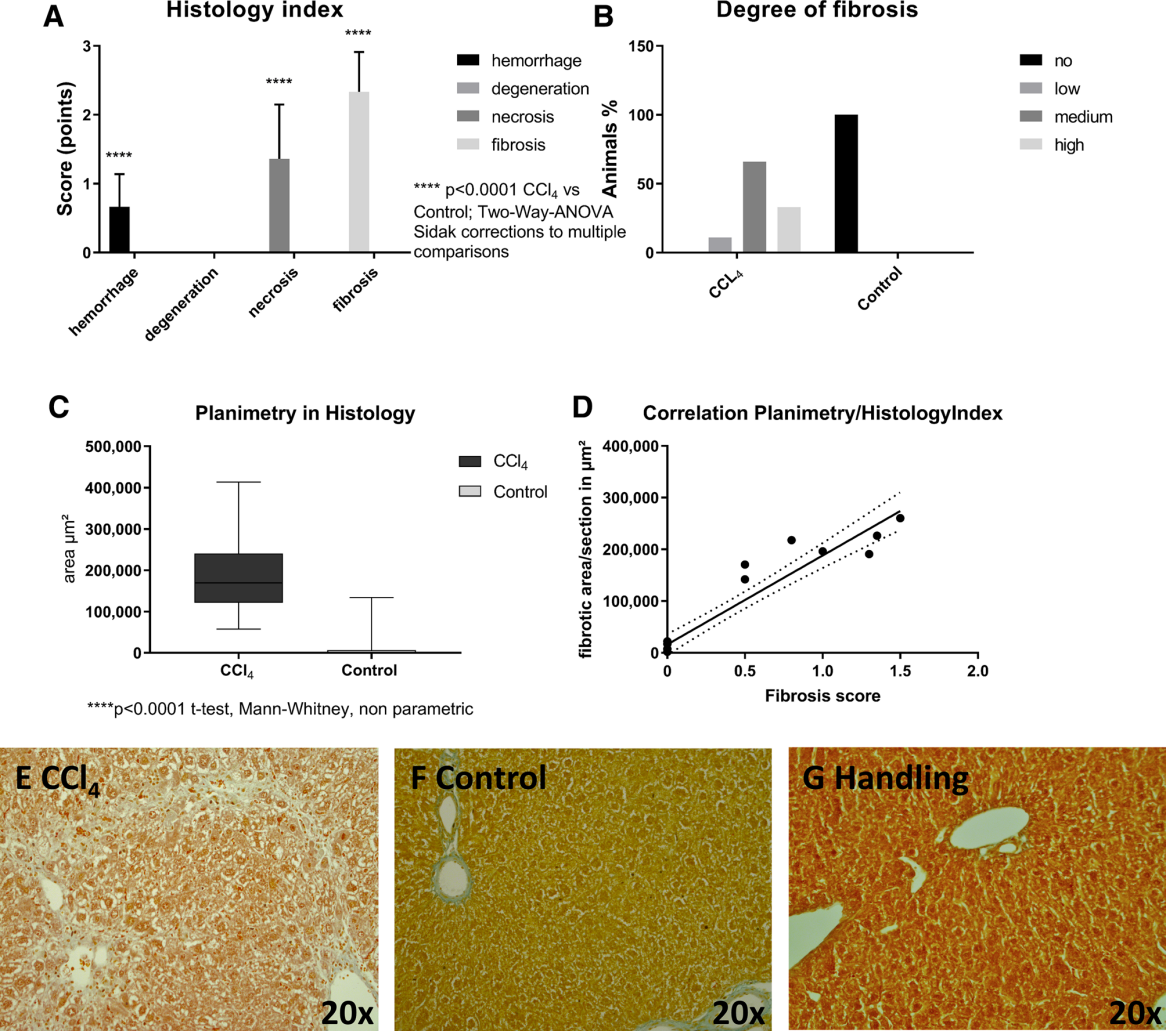
Histological analysis of liver tissue after 4 weeks of treatment (CCl_4_, control) and microscopic images of liver sections (CCl_4_, control, handling) in Masson Goldner staining (× 20). Collagen deposits are indicated as pale blue colored tissue (**A**) Histology index. Two-way ANOVA F(3, 304) = 142.6 ****p < 0.001(Sidak corrections for multiple comparisons) p_adj_ =  < 0.0001. (**B**) Degree of fibrosis. (**C**) Planimetric analysis. ****p < 0.0001 t-test, Mann–Whitney, non-parametric. (**D**) Correlation of planimetry and histology index. r = 0.95; r^2^ = 0.91; 95% confidence interval 0.8729 to 0.9834. **(E)** Representative image of a liver section showing moderate fibrosis in the CCl_4_ group (**F**) a section obtained from a control animal and **(G)** histological appearance of liver section obtained from a handling group animal. The graphs were generated using GraphPad Prism version 7.00 for Windows (GraphPad Software, La Jolla California USA, www.graphpad.com).

Histopathological evaluation show a clear fibrosis of the liver tissue with slight hemorrhagic and necrotic sections in the CCl_4_ treatment group. All animals in the treatment group showed liver fibrosis in the histopathological examination (Fig. 2B,E). Due to the lack of histopathological and macroscopic differences between the control and the handling group, the handling group is not presented.

The quality of fibrosis was moderate in most cases (66.67%), while in 22.22% of the animals it was severe. In 11.11% low liver fibrosis was noticed. The planimetric evaluation of fibrotic areas within the liver slices showed distinct hepatic fibrosis within the treatment group that correlated well with the histology index of the histopathological examination (r^2^ = 0.9092) (Fig. 2C,D). Histological results are shown exemplarily in Fig. 2E,F.

**Figure 3:**
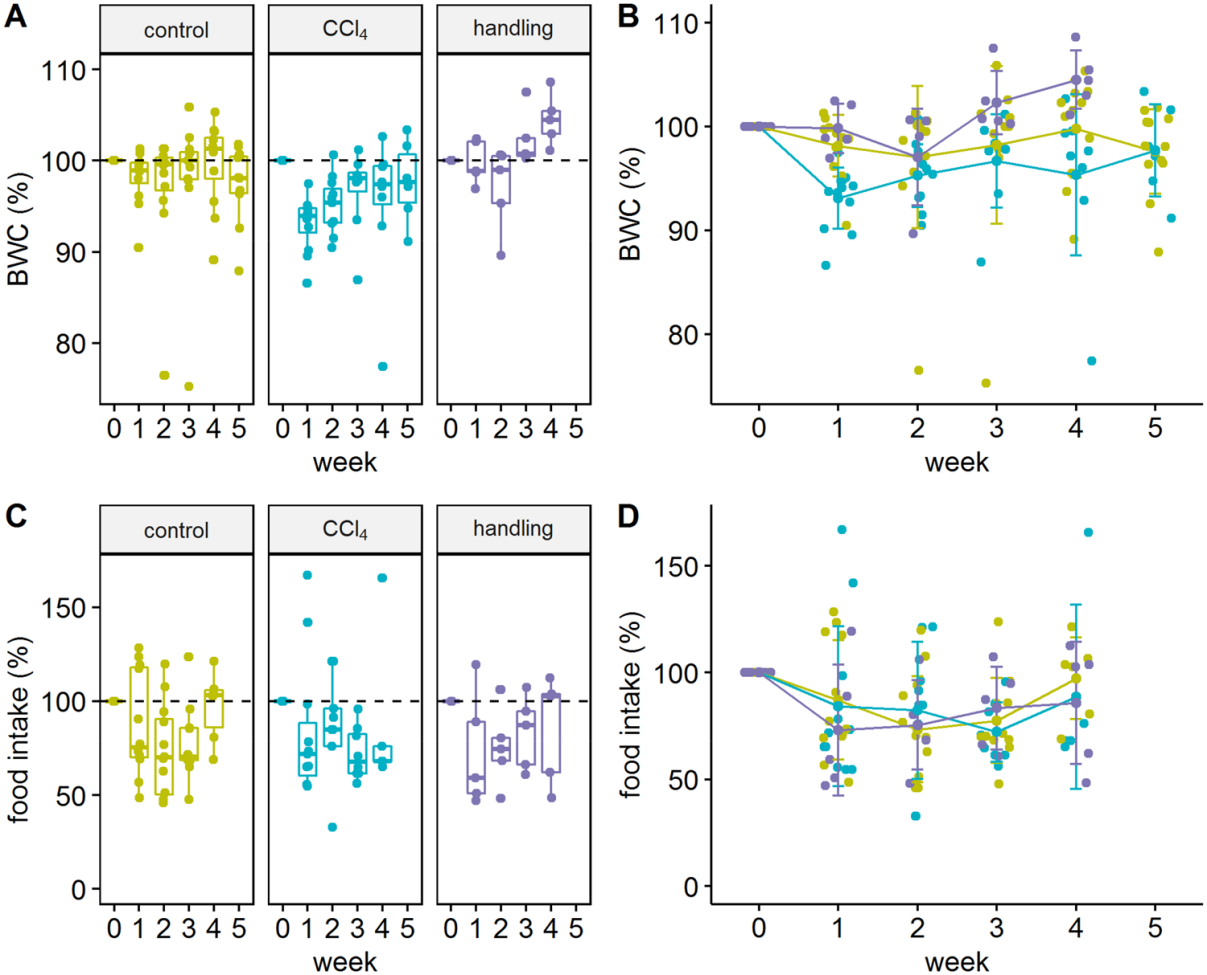
Weight development and food intake over the experimental period. In the handling group no weight measurement was performed on the day of sacrifice (**A**) Values for body weight are shown as boxplots. (**B**) Comparative body weight changes (BWC) in % with a representation of individual values over time. Type III ANOVA F(2; 27.72) = 3.581; week 1 CCl_4_ vs. control * p < 0.05 post-hoc (Tukey test) p_adj_ = 0.016; week 1 CCl_4_ vs handling * p < 0.05, p_adj_ = 0.013; week 4 CCl_4_ vs. handling ** p < 0.01, p_adj_ = 0.001. (**C**) Boxplot of group values for food intake. (**D**) Comparative food intake changes between the treatment groups. The graphs were generated using the R software version (3.6.2) (R Core Team (2017). R: A language and environment for statistical computing. R Foundation for Statistical Computing, Vienna, Austria. https://www.R-project.org/).

The initial body weight was 25.8 ± 1.41 g for the CCl_4_ group, 26.1 ± 1.40 g for the control group, and 26.62 ± 1.0 g for the handling group, respectively. The weight development within the experiment showed a significant weight loss within the first week of treatment of animals treated with CCl_4_, while the weight loss was only moderate in the control and in the handling group. However, food intake and body weight changes did not correlate (r^2^ = 0.15). After the second week of the experiment, no significant differences between control and CCl_4_ groups were observed, but the handling group showed significantly more weight gain compared to the treatment group (Fig. 3B).

**Figure 4:**
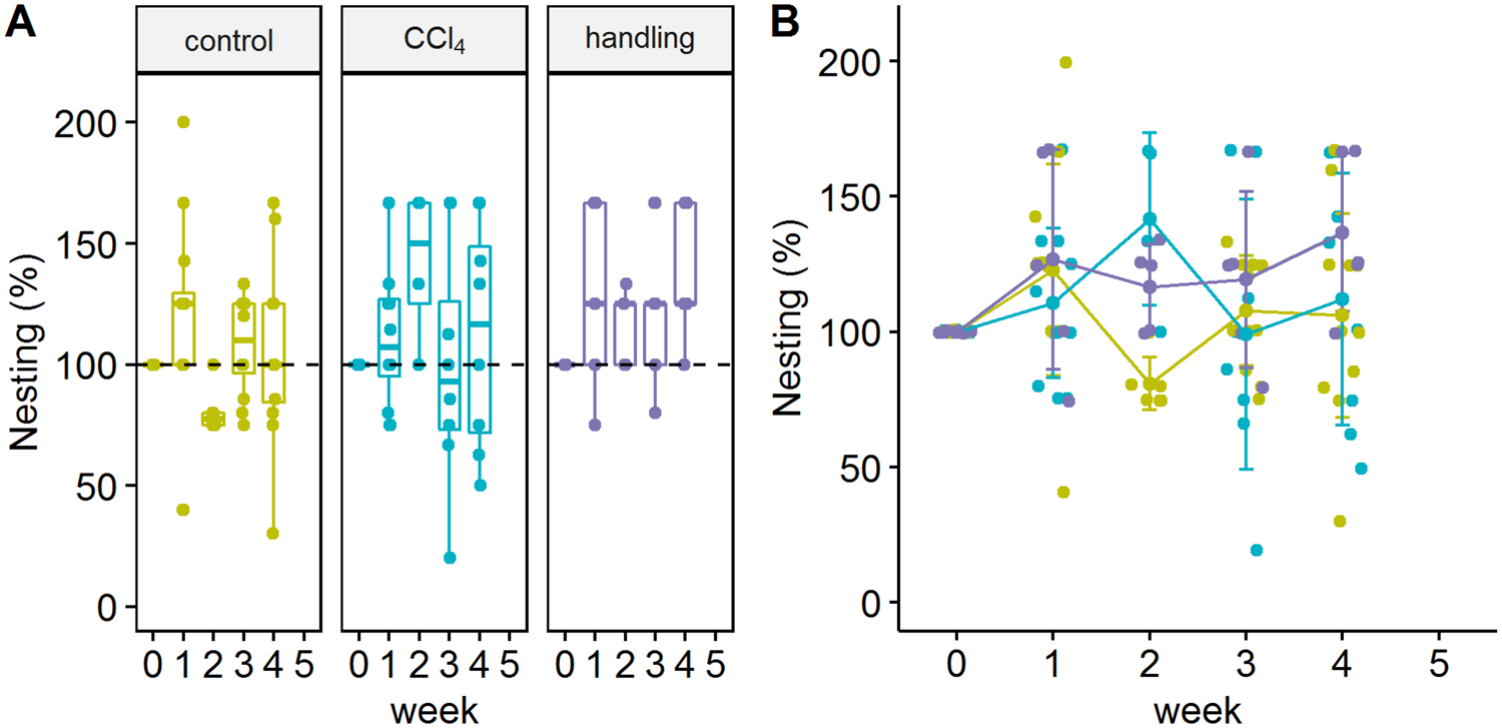
Nesting quality. (**A**) Boxplot display for the nesting quality in the three groups. (**B**) Single value representation of all study groups in % of the nest building behavior over time F(2;26.72) = 1.0260, week 2 CCl_4_ vs. control *p < 0.05 p_adj_ = 0.026. The graphs were generated using the R software version (3.6.2) (R Core Team (2017). R: A language and environment for statistical computing. R Foundation for Statistical Computing, Vienna, Austria. https://www.R-project.org/).

Significant differences in nest building quality between the treatment and control groups were identified in the examination of nest building behavior in the second week of treatment (Fig. 4A,B). No significant difference between the experimental groups and their baseline values could be detected. Animals of the CCl_4_ group showed an increased nest-building score compared to the control group in the second week of treatment. Nevertheless, no significant prolongation of the first interaction of the animals with the nesting material (latency to start nesting) could be detected. The nest-building score throughout the complete duration of the experiment was on average 4.02 ± 0.43 in the CCl_4_ group, 4.09 ± 0.38 in the control group, 4.44 ± 0.80 in the handling group. However, all animals consistently showed a good nest building behavior during the experimental phase with a trend of the handling group in the fourth week of the experiment to increase compared to the baseline measurement.

**Figure 5:**
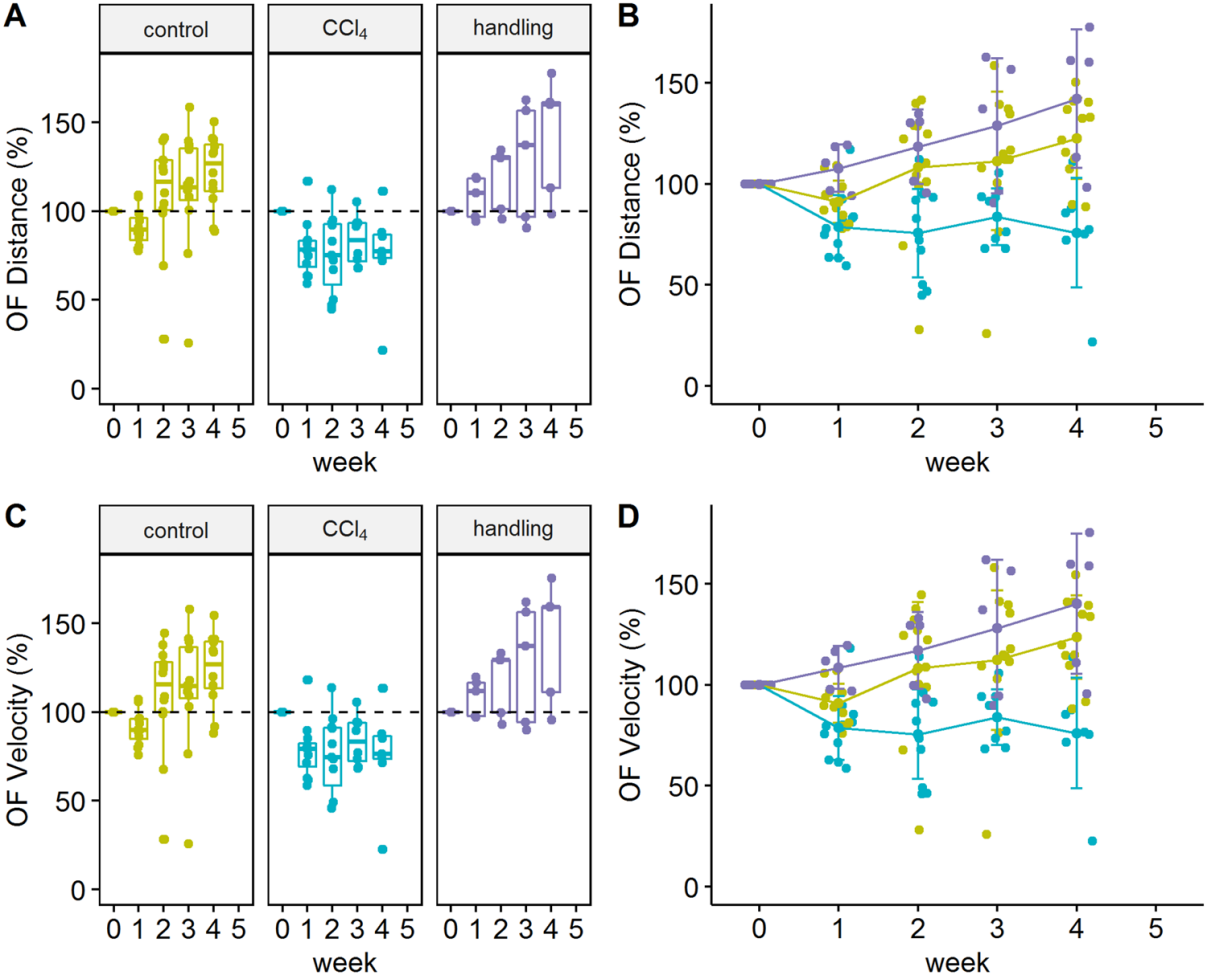
Analysis of movement modalities in the Open field test. (**A**) Boxplot of the distance changes of the two experimental groups (**B**) Percentage change in distance of the two experimental groups over time F(2; 26.53) = 13.58; CCl_4_ vs. control week 2 and 3 p < 0.01** p_adj_ = 0.001 for week 2 and p_adj_ = 0.005 for week 3; week 4 p < 0.001***; CCl_4_ vs. handling week 1 p < 0.05** p_adj_ = 0.03; week 2, 3 and 4 p < 0.001***; control vs. baseline n.s.; control baseline vs. week 4 *p < 0.01 p_adj_ = 0.005; CCl_4_ baseline vs. week and 1, 2 and 4 **p < 0.01 p_adj_ = 0.01 for week 1, p_adj_ = 0.003 week 2 and p_adj_ = 0.004 for week 4. (**C**) Boxplot of velocity changes in the three experimental groups. (**D**) Changes in velocity over time F(2;26.52) = 13.15; CCl_4_ vs. control week 2 and 3 p < 0.01**, p_adj_ = 0.001 for week 2 and p_adj_ = 0.004 for week 3; week 4 p < 0.001***; control baseline vs. control week 4 **p < 0.01 p_adj_ = 0.004; CCl_4_ baseline vs. week 1,2 and week 4 *p < 0.01 p_adj_ = 0.009 for week 1, p_adj_ = 0.003 for week 2 and p_adj_ = 0.005 for week 4; CCl_4_ baseline vs. week 2 **p < 0.01 p_adj_ = 0.003 for week 2; handling baseline vs. week 3 *p < 0.05, p_adj_ = 0.03, week 4 **p < 0.01, p_adj_ = 0.001. The graphs were generated using the R software version (3.6.2) (R Core Team (2017). R: A language and environment for statistical computing. R Foundation for Statistical Computing, Vienna, Austria. https://www.R-project.org/).

The analysis of the Open field data showed no significant differences in the distribution of the animals in the defined area (corner, middle, or border zone; data not shown). However, from the second week of treatment until the end of the experiment, significant differences were observed between the treatment and control group, both in the distance of movement and in the overall velocity of the animals (Fig. 5). Differences between the handling and control group were not significant. However, for both overall distance and velocity the handling group showed a trend towards higher performance.

Fecal corticosterone metabolite (FCM) analysis and Rotarod performance (data not shown) showed no significant differences between the experimental groups and their baseline values during the time of the experiment. In the second week of treatment, the animals in the CCl_4_ group burrowed fewer food pellets than the oil-treated control group (data not shown). However, due to a lack of showing burrowing behavior in enough animals in the handling group, no full statistical analysis between these groups could be performed.

**Figure 6:**
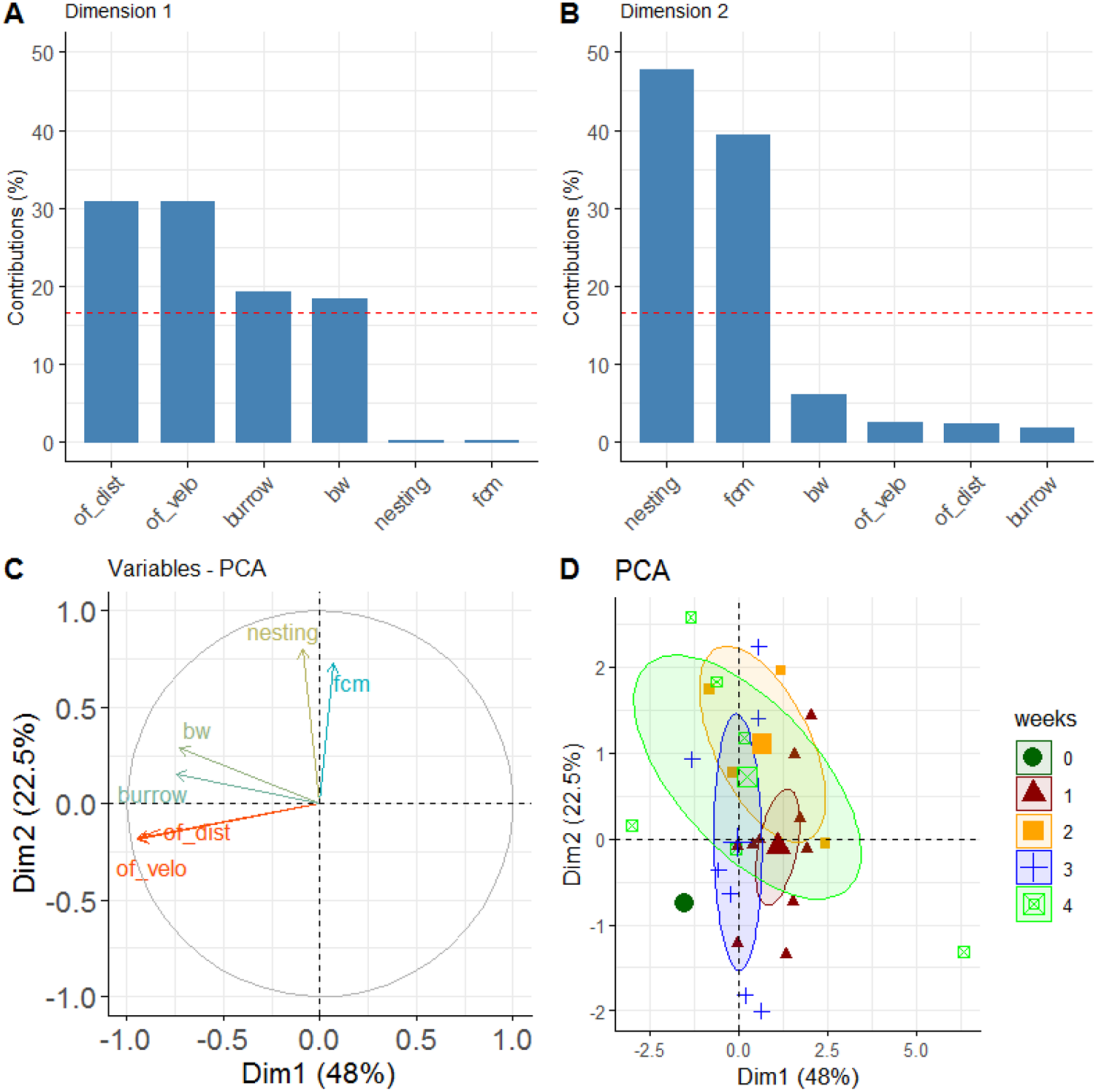
Principal Component Analysis of the CCl_4_ group. (**A**) Variance contributions of factors in % in the first dimension. (**B**) Contribution of factors in % in the second dimension. (**C**) Variable correlation plot showing the relationships of all variables in the experiment. Positively correlated variables are grouped, negative ones show in different directions. The vector lengths represent the quality in the factor space. The longer they are the better the variables are represented in the shown dimensions. (**D**) Projection of individual animals into the two-dimensional PCA factor space. Group centroids are characterized by the 95% confidence ellipses and the experimental times are color-coded as weeks. The graphs were created using the R software version (3.6.2) (R Core Team (2017). R: A language and environment for statistical computing. R Foundation for Statistical Computing, Vienna, Austria. https://www.R-project.org/).

Principal Component Analysis (PCA) was performed to investigate the relevance of the different measurement parameters in the experiment (Fig. 6). It revealed that the Open field test, both in distance and velocity, is the main factor in terms of variance contribution relevant to the CCl_4_ group (Fig. 6C). The PCA for the control group showed a similar factor distribution with an additional stronger contribution for the burrowing behavior (data not shown).

**Figure 7:**
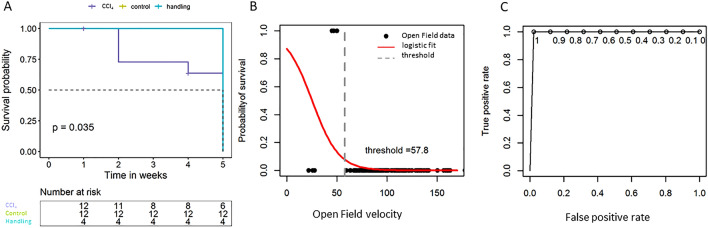
Survival curve of the animals **(A)** and threshold detection and optimization for Open field velocity data with regard to the animal survival rate. **(B)** Logistic regression and model fit (red line) for threshold definition using binary-labeled (censored) Open field velocity data. Animals showing values below the threshold have a higher likelihood of dying. **(C)** The ROC curve for the optimized threshold shows good discrimination between survivors and non-survivors in the logistic model (AUC = 0.89). The graphs were generated using the R software version (3.6.2) (R Core Team (2017). R: A language and environment for statistical computing. R Foundation for Statistical Computing, Vienna, Austria. https://www.R-project.org/).

The animal survival rate was determined via a Kaplan–Meier estimator. The curve showed a median survival rate of only 50% of the CCl_4_-treated group and 100% in the control group (Fig. 7A). An increased mortality rate was observed in week two in the CCl_4_ treatment group (n = 3). Among the six deceased animals, n = 4 died and n = 2 animals had to be euthanized, because they reached the humane endpoint. These animals are marked as censored with a dash in Fig. 7A. At the beginning of week 5, the remaining animals were euthanized according to the protocol.

The variables were analyzed by univariate Cox-regression ^7^ resulting in a hazard ratio (HR) of 1.2 (95% confidence interval 0.86–1.6) for the candidate predictor variable Open field velocity. The threshold for predicting mortality was optimized by logistic regression and receiver operating characteristic (ROC) analysis (Fig. 7C). The optimal cutoff for predicting death was found to be at 57.8% in Open field velocity with an odds ratio of 4.57 (Fig. 7B).

## Discussion

To investigate the severity assessment of a model of CCl_4_-induced liver damage, different methods were used in this study to assess the severity of this model. These included both clinical parameters and behavioral test methods. The development of fibrosis is based on the toxicity of CCl_4_ in the liver metabolism. During the metabolism of CCl_4_, radicals are formed, which have a hepatotoxic effect and cause damage and necrosis of hepatocytes^8^. A cascade is initiated, in which hepatic stellate cells become activated and produce excessive collagen deposits in the extracellular matrix, and thus leads to hepatic fibrosis^9^. Fibrosis can be described as a kind of scar tissue formation and a repair mechanism of the liver for toxic substances. While fibrosis is often clinically mild to moderately apparent, these mechanisms can lead to cirrhosis, which is associated with a deformation of the liver structure and liver failure^10^. For this reason, experimental models of liver fibrosis in mice are of great importance in biomedical research.

An ideal model for the investigation of fibrosis should, therefore, be able to differentiate fibrosis in the same facets as in humans, while being as less harmful as possible to the model organism, the mouse. In our study, the main focus was to produce CCl_4_-induced moderate fibrosis. The 4-week CCl_4_ injection model, which was chosen for this study because it is widely used to induce moderate fibrosis, was performed according to a well-established Standard Operation Procedure^11^.

With the histopathological results of this study, we could demonstrate that this model is capable of inducing stable fibrosis in C57BL/6N mice (Fig. 2). All animals in the treatment group showed fibrosis, which was expressed as moderate in 66.67% of the cases. Both, planimetric evaluation and histopathological scoring showed a good correlation. Typically, a manual histopathological examination requires a lot of experience and involves a certain risk of subjectivity, while planimetric evaluation can be performed with a high degree of standardization and with little dependence on the observer. Therefore, planimetric evaluation is easier to use and more reproducible. For fibrosis staging of renal tissue, even automated examination methods on a planimetric basis are available^12^.

To assess the severity of the CCl_4_ model, we examined three different rational approaches: (i) the actual stress caused by CCl_4_ administration, taking into account acute toxicity and pain; (ii) chronic effects of cumulative treatment (e.g. multiple injections and chronic toxicity); (iii) survival rate and determination of cut-off values in behavioral testing to better estimate the risk of mortality.

In such studies, it is also necessary to relate the metabolism of CCl_4_ and the pathogenesis of fibrosis to these observations, which can also be divided into three distinct stages, namely (i) acute hepatic injury, (ii) first accumulation of fibrotic tissue, and (iii) stable fibrosis^5^. The acute metabolic toxicity of CCl_4_ injections in the liver occur about 48 h after administration^13^. By repeated injections 72 h intervals, a continuous toxicity wave can be established for the development of fibrosis. The development of fibrosis evolves after 2 weeks of treatment and manifests itself as stable fibrosis after 4 weeks^5^.

This progression was also supported by our results. With the first injections of CCl_4_, the acute stage and acute stress development phase is initiated, which is indicated by differences in body weight development (Fig. 3A,B) and Open field performance (Fig. 5). The animals subjected to CCl_4_ injection showed a significant body weight reduction in the first week compared to the control group animals. The development of body weight change in CCl_4_ treated animals compared to the handling group showed significant differences in week 1 and week 4. This was adjoined with a trend to gain more weight in the handling group, while there was no deviation in food intake in all of the three groups detectable. Therefore, weight reduction most likely results from increased metabolism through pain or stress in the first week of treatment when the stress situation caused by the treatment first affects the animals and no adaptive mechanisms can occur. Although the animals of the CCl4 group show a slight increase in body weight in the following weeks after the initial weight loss of week 1, most likely due to a habituation effect, they do not reach their initial weight.

Previous studies have shown that burrowing and nesting behavior are luxury behaviors that can serve as indicators of stress or pain. They are not essential for survival, but are the first features changing when the general condition decreases^14,15^. Consequently, they are particularly suitable for assessing acute stress. No significant change in nesting behavior was noticed in the acute phase of the first week. However, the nesting behavior regarding nest quality was increased in the second week of treatment in the CCl_4_ group with a trend of increase in the control group. For rodents, nest-building is not only a marker of well-being, but also serves for heat conservation and as a shelter from predators^16^. This implies that increased nest-building quality can indicate an increased need for protection, but also show wellbeing. This notion is supported by the results of the handling group which, not significantly different from the control group, are lying between both, the CCl_4_ and control group regarding the performance of nesting behavior. We conclude from all the other investigations of this study that the results of the handling group consistently trend to show a lower severity level, compared to the control group and significantly to the treatment group also shown in AST levels (Fig. 1), mortality, and differences in Open field performance (Fig. 5).

In the second week of CCl_4_ treatment, liver damage is reflected in remodeling processes and correlated with the chronic phase of stress in the animals. However, it is difficult to classify the model into ‘mild’, ‘moderate’, or ‘severe’ severity, based on the results of behavioral characteristics. These parameters do not provide an exact measure of the severity since behavioral tests can vary between individuals and therefore show higher background noise levels^17^.

This study was classified as mild to moderate in the prognostic severity assessment as defined in Annex VIII of the 2010/63 EU Directive^1^. The individual components, e.g. injections, behavioral test or individual posture, were found to be of mild severity, while the accumulation of treatment over time was found to be of medium severity. This classification is supported by the fact that we can record few deviations in the luxury behavior, e.g. in the acute phase or at the beginning of the chronic phase, but these changes in vital basic values, such as weight development or food intake, showed no severe differences (Fig. 3).

Contrary to this, there was an overall high rate of 50% lethality in the CCl_4_ group (Fig. 7A). This indicated a severe procedure for 33.33% of the animals, which died during the study, while 16.67% of these animals had to be sacrificed due to compliance with humane endpoints and therefore could be protected from more than moderate exposure. Therefore, we aimed not only to evaluate the detection of exposure, but also to characterize which parameters are most suitable for monitoring severity in the treated animals. Here, the treated group was in the focus of examination, since the control group and the handling group had no mortality risk during treatment. However, our results show that handling and behavior tests seem not to have negative effects on the severity of the examinated animals. The PCA for the CCl_4_ animals showed that the performance in the Open field is the most important factor in the investigations (Fig. 6). To assess the risk of mortality, the main contributing variables to PCA were further studied by univariate Cox-regression to calculate the individual hazard ratio on animal survival. We could demonstrate that the monitoring of the Open field velocity is a suitable measurement parameter for the probability of survival, in which a velocity below the defined threshold of 57.8% corresponds with a 4.75-fold higher risk of mortality.

## Conclusions

The severity of the CCl_4_ model in mice tends to show a moderate severity level. However, it was associated with an increased mortality (33.33%) in our study. Although the administration of CCl_4_ is a long-known and widely used model, the publication rate of survival and studies on the mortality of these study models are very rare. However, the severity level of an animal model can most likely also depend on strain, genetic background, injection interval, or dosage. Therefore these measures should be strategically investigated and verified for their refinement potential in future studies. To prevent increased mortality rates, the measured parameters selected here, especially the Open field test, can be used in estimating severity and animal monitoring. The analysis for the survival prediction in this study is not unrestricted. The definition of the threshold was based on two criteria, mortality and Open field performance, which had to occur on the same day. Nevertheless, the ROC AUC analysis with the value of 0.89 showed that this test procedure is suitable for the assessment of mortality and the definition of a cut-off value for severity assessment. However, this procedure should be validated and refined in further studies. In sum, the study demonstrates the general feasibility of assessing severity using behavioral tests and could demonstrate that the Open field test is a possible candidate parameter for risk assessment. Furthermore, this study also shows that the systematic investigation of severity levels and mortality rates should be investigated to develop sufficient refinement measures for experimental models.

## Materials and methods

### Ethical statement

This animal study was planned and performed following the EU Directive 2010/63 and the Federal German law regarding the protection of animals^1,18^. The proposal of this animal study was approved by the Governmental Animal Care and Use Committee (LANUV, North Rhine-Westphalia, Germany, AZ: 84-02.04.2014.A417) and conducted in compliance with institutional guidelines as well as The Guide for The Use of Laboratory Animals^19^. The present study was designed, performed, and reported according to the principles of the ARRIVE (Animal Research: Reporting of In Vivo Experiments) guidelines^20^.

### Animals

For the animal number calculation, a power analysis was performed using the G-Power program^21^ based on assumed differences between the experimental groups. An animal count of at least nine animals per analysis group was considered as the minimum group size. The animals were kept in self-constructed observation cages (Poly-methyl methacrylate (PMMA)) 30 × 30 × 40 cm (W × D × H) in a specific-pathogen-free (SPF) barrier controlled according to FELASA guidelines^22^. Humane endpoint criteria were established for all animals. These endpoints were applied when > 20% body weight loss, abdominal enlargement, vocalization during grasping, lethargy, or hypothermia occurred.

### Study design/experimental procedure

**Figure 8:**
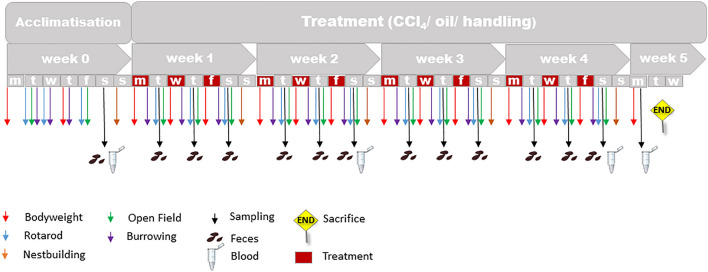
Study design with all procedures and treatments. In week 0, training and baseline measurements were performed. At the beginning of week 5, the animals were sacrificed.

In this study, 29 male C57BL/6N mice weighing 25.9 ± 1.4 g were single-housed and treated three times per week for four consecutive weeks with either i.p. injection of 0.6 ml/kg body weight CCl_4_-mixed germ oil (Mazola Keimöl, Peter Kölln GmbH & Co. KGaA, Elmshorn, Germany) solution (treatment group n = 12), only germ oil as vehicle (control group n = 12) or handled (handling group n = 5) as to be injected. All liquids were initially tested negative for bacterial content and pyrogen detection. The injection solutions were given only at room temperature with a volume of 50 µl/animal/injection. The animals in the handling group were taken out of the cage handled and restrained in the same way as injected and subsequently returned to the cage without injection. Treatment (injection or handling only) was performed on Monday, Wednesday, and Friday in the morning between 9:00 and 9:40 a.m. (Fig. 8). Severity assessment was conducted with the evaluation of weight gaining profile, behavioral or locomotion tests, and evaluation of serum parameters. All tests and evaluations were conducted on the same daytime during the follow-ups. Training and baseline measurements were conducted for each part of the experiments one week before the start of the injections. The chronological sequence of the different test procedures is depicted in Fig. 8. Histopathological examinations for differentiation and classification of fibrosis were conducted retrospectively. At the end of the experiment, the animals were euthanized under deep anesthesia (Xylazine 10 mg/kg + Ketamine 100 mg/kg i.p), exsanguinated, and the abdominal cavity opened for organ removal. Handling, injections, and behavioral testing were always performed at the same daytime by the same experimenters, who have had > 3 years of experience in laboratory animal science.

### Data collection and measurement methods

#### Weight profile, food, and water intake

Body weight was measured initially before allocating the animals to the different treatment groups (CCl_4_/oil/handling). All animals were allocated randomly according to the average starting weight of both experimental groups. Water and food (ssniff Spezialdiäten GmbH, Soest, Germany) were obtained ad libitum, replaced and weighed once a week.

### Nesting behavior

The nest building behavior was determined once a week. For this purpose, a nestlet (Plexx B.V., AB Elst, The Netherlands) was placed in the middle of the cage for each animal after the weekly change of cages. Subsequently, the animal was filmed for the next 23 h. Thereafter, the quality of the nest is determined based on Deacon's evaluation score on nest building into five different nest qualities^23^. In brief, the nest was evaluated as follows: score of 1 means that the nestlet is more than 90% untouched and a score of 5 equals a perfect built nest. Additionally, the time until the first contact with the nestlet (latency to start) was determined based on the video analysis.

### Rotarod performance test

Rotarod testing was performed with TSE Rotarod (Rotarod Advanced, TSE Systems, Bad Homburg, Germany) system three times per week, one day after each injection. All animals were trained two times before the test. A third rotarod testing was performed to set baseline values for each animal. One rotarod test contained three cycles with 120 s/cycle split into two phases of 60 s. The frequency in the first 60 s was set to 10 rotations/min and in the second 60 s the frequency increased up to 20 rotations/min.

### Open field test

The Open field test was performed using a custom made Open field 72 × 72 × 50 cm (WxDxH). The Open field arena was made of grey matt PMMA. The floor was laminated with a whitish, light-colored foil, on which the marching lines of the Open field squares (24 pcs. a 16 × 16 cm) drawn under the foil could slightly shine through. The animals were tested one day after i.p. application (Fig. 8). Therefore, the mice were placed in the middle of the Open field and videotaped with a digital video camera (Basler Camera GigE color 1/1.8", Lens Std CS mount 4.5–12.5 mm 1/2", Basler AG, Ahrensburg, Germany) and the Noldus System (Noldus, Wageningen, The Netherlands). The Open field was cleaned, disinfected with antifect N liquid (Schülke & Mayr GmbH, Norderstedt, Germany) and wiped off after each animal testing to avoid chemo-communication via feces or urine. Evaluation of velocity, distance moved, and residence time was performed using Noduls Ethovision XT 12.1 software.

### Burrowing

The Burrowing test was always performed three hours before the start of the dark phase (i.e. at 4 p.m.) three times a week on the day animals received CCl_4_, oil injections or handling. Baseline measurements were carried out twice during the acclimatization week. The burrowing behavior was tested according to the set up by Jirkof et al.^14,15^. In brief, a bottle of counted food pellets (60 pcs) and an empty bottle was placed in the cage. The animals were filmed for 16 h until the next morning. The weight and the number of moving pellets were evaluated then off-line.

### Analysis of fecal corticosterone metabolites (FCMs)

Fecal samples were always collected by physiological defecation during handling for the open field and rotarod testing at the same daytime within the experiment three times a week on the day after CCl_4_ application. FCMs were analysed with a previously published and validated enzyme immunoassay^24,25^.

### Serum analysis

Blood samples were taken every 2 weeks by retro-bulbar blood sampling under isoflurane anaesthesia after the performance of the behavioral tests. At the end of the observation period, exsanguination was performed in terminal anaesthesia by direct cardiac puncture using a 22G needle. Serum AST levels were measured with the VITROS 350 Integrated System Analyzer (Ortho-Clinical Diagnostics GmbH, Neckargemünd, Germany).

### Histopathological evaluation

For histopathological examination, livers were fixed in a 4% formaldehyde solution for 48 h, drained, and embedded in paraffin. Subsequently, slices were prepared and stained. Staining was performed in both hematoxylin & eosin (HE) and Masson Goldner staining. The slides were examined and evaluated by a senior pathologist blinded to the experimental conditions for the presence of inflammation, necrosis, fibrosis, granuloma formation, degeneration and hemorrhages. The grading was evaluated according to the Desmet scheme for fibrosis classification. (0 = no fibrosis, 1 = low fibrosis, 2 = moderate fibrosis, 3 = high fibrosis)^26^. To quantify the degree of fibrosis, the stained areas in the histological samples were measured with the help of the software Image J (National Institutes of Health, Bethesda, MD, USA).

### Survival

The survival rate of the animals was recorded and displayed using the Kaplan–Meier analysis in the R “survival” package^27^. Animals that had to be euthanized during the experiment due to reaching the humane endpoint were reported as censored. The Hazard Ratio for each variable was extracted from the coefficients of a univariate Cox-regression. Censored Open field velocity data were dummy coded as 0 (survivor) and 1 (non-survivor) to serve as dependent variables in a binary generalized linear model with the logit link function. The resulting probabilities were determined in threshold (in 0.05 steps) to find the best discrimination between classes. The winning set determined the threshold and was used in displaying the ROC curve using the “ROCR” package^28^.

### Statistical analysis

Data were analyzed with GraphPad PRISM 7 software (GraphPad Software Version 7, La Jolla California USA) and free software for data manipulation, calculation, and graphical display (R environment)^29^. Prism was used for the statistical evaluation of the histopathological results, a two-way analysis of variance (ANOVA) was carried out using the Sidak correction for multiple comparisons. For single statistical investigations (e.g. planimetry) between the two groups a t-test or non-parametric Mann–Whitney U-test was performed. Due to inter-individual ranges, all test results were normalized to their baseline values.

For the analysis of individual experimental variables, a linear mixed-effects model was fit for each group (control, handling, and CCl_4_) in an interaction with time (week) for the fixed effects (“lme4”)^30^. Animal ids were included as random effects. From the individual fits, type III ANOVA error tables were produced and estimated marginal means (“emmeans”) were used for building contrasts^31^. Depending on the nature of the contrast this included Tukey’s test for multiple comparisons, Sidak correction, or the Dunnett test. Principal component analyses (PCA) for the CCl_4_ and control group were performed using the “factoextra” package^32^. The Figs. 1, 3, 4, 5, 6 and 7 were created with the R software (3.6.2)^29^ using the basic plot function, or the following packages for visualization: ggplot2^33^, ggpubr^34^, survminer^35^ and factoextra^32^. Visualisation of Fig. 2 was performed using GraphPad PRISM software version 7.0, design and visualization of Fig. 8 war performed using PowerPoint version 2019.
